# An Ethnobotanical Study of the Medicinal Plants Used as Anti-Inflammatory Remedies in Gampaha District, Western Province, Sri Lanka

**DOI:** 10.1155/2018/9395052

**Published:** 2018-06-03

**Authors:** Mayuri Tharanga Napagoda, Thamudi Sundarapperuma, Diroshi Fonseka, Sachinthi Amarasiri, Prabath Gunaratna

**Affiliations:** ^1^Department of Biochemistry, Faculty of Medicine, University of Ruhuna, Galle 80000, Sri Lanka; ^2^Allied Health Science Degree Programme, Faculty of Medicine, University of Ruhuna, Galle 80000, Sri Lanka

## Abstract

The application of traditional medicinal plants as anti-inflammatory remedies has been practiced in Sri Lanka for thousands of years. Although there is a rich reserve of indigenous knowledge of medicinal plants, the preservation and the scientific validation of these claims are still in its infancy. Thus, the study was carried out in one of the administrative areas of Sri Lanka known as Gampaha District to assess the significance and contribution of medicinal plants in inflammatory conditions. The data were collected through semistructured and open-ended interviews from 458 volunteers. Ethnobotanical data were analyzed using the relative frequency of citation (RFC), family importance value (FIV), and use value (UV). Out of the total participants, 50.7% claimed the use of medicinal plants for the treatment of inflammatory conditions such as fever, cough, asthma, swellings, and pain in the joints. A total of 43 medicinal plants belonging to 28 plant families were mentioned, out of which *Coriandrum sativum* (RFC = 0.23) was the most cited species. The most cited plant family was Fabaceae, and the family importance value was highest in Apiaceae. The majority of the nonusers of the herbal remedies mentioned that they would shift to herbal products if scientific information is available on the efficacy of these products.

## 1. Introduction

Inflammation is a pathophysiological response to injury, infection, or irritants and is characterized by heat, redness, pain, swelling, and disturbed function of the organs. Since ancient times, inflammatory disorders and related diseases have been treated with plants and plant-derived formulations [1]. Over the last two decades, a significant amount of evidence has emerged indicating that chemically diverse classes of plant secondary metabolites are of potential interest for therapeutic interventions in several inflammatory diseases. A number of studies revealed the ability of plant extracts or plant secondary metabolites to control the levels of various inflammatory cytokines or inflammatory mediators including IL-1, IL-6, IL-10, TNF-*α* [2], NF-*κ*B, NO, iNOS, COX-2 [3], and 5-LO [4].

Plants and their products have been systematically used in Sri Lanka for treating illnesses for over a thousand years. Among the native flora of Sri Lanka, more than 1400 plants are used in indigenous medicine [5], and a large number of plants are extensively used to alleviate the pathological conditions associated with inflammation [6]. However, there has been a dearth of published information on ethnobotanical studies on anti-inflammatory remedies within the Sri Lankan context.

Four systems of traditional medicine have been adopted in Sri Lanka: Ayurveda, Siddha, Unani, and Deshiya Chikitsa. The Ayurveda and Deshiya Chikitsa systems use mainly plant and herbal preparations for the treatment of diseases [7]. Different regions of the country have their unique traditional medicine systems (known as Deshiya Chikitsa) and practitioner pedigrees. Out of these different regions, Gampaha District is considered as the home for several well-known traditional practitioners in the country. Although the modern health care facilities are readily available in this area, traditional medicinal practices are quite popular and widely accepted by the people. Ten Ayurvedic hospitals governed under the Ministry of Indigenous Medicine are located within the district to meet this demand [8]. Therefore, the study area for this research has a rich potential for utilization and consumption of medicinal plants. However, an in-depth study has not been pursued yet to assess the significance and contribution of medicinal plants/herbal therapeutics to the day-to-day life of the inhabitants of Gampaha District. In order to fill this gap in knowledge, the traditional medicinal uses of plants for inflammatory conditions have been documented in the form of an ethnobotanical inventory in order to assess the popularity and usage of medicinal plants in the study area.

## 2. Materials and Methods

### 2.1. Study Area

**Figure 1:**
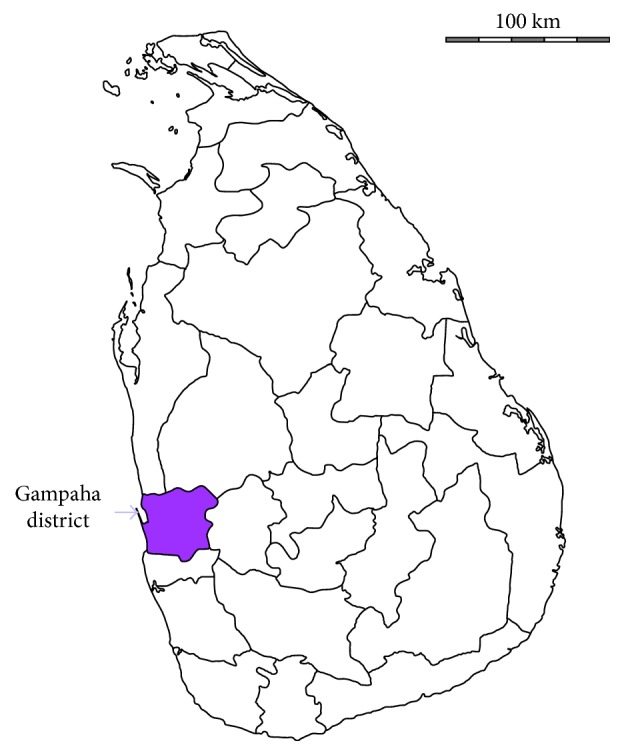
Location of Gampaha District.

Gampaha District is located in the Western Province of Sri Lanka (Figure 1) and has an area of 1,387 km^2^. The district is divided into 13 divisional secretariat divisions, which are further subdivided into 1,177 *grama niladhari* divisions. There are 1,784 villages, and the total population of the district is reported as 2,280,860 [9]. According to the geographical location, this district belongs to the coastal plain and shows plain geographical characteristics in most of the areas and mountainous geographical characteristics in northern and eastern parts. The forest coverage of the district is estimated as 240.8 ha, and the district contains three isolated natural forest patches: Horagolla National Park, Maimbulkanda sanctuary, and Yakadawala forest reserve [10].

### 2.2. Ethnobotanical Field Survey and Data Collection

Ethnobotanical information in the study area about the use of plant species for the treatment of inflammatory conditions was documented from all thirteen divisional secretariat areas, that is, Attanagalla, Mirigama, Minuwangoda, Gampaha, Mahara, Dompe, Ja-Ela, Divulapitiya, Katana, Biyagama, Negombo, Wattala, and Kelaniya. This survey was carried out from December 2014 to December 2016, and the data were collected through semistructured and open-ended interviews using a pretested questionnaire. The questionnaire was pretested by administering to a selected group of the population with a similar sociocultural background in different administrative districts of Sri Lanka. The random sampling method was used to recruit 458 volunteers from the general population of the district who were aged above 30 years. The participants were selected randomly from a list of households in each divisional secretariat area, and visits were made to each of those households for data collection. Written informed consent was obtained prior to the study. The number of informants for a species mentioning its uses was assessed and categorized. The questionnaire used to compile ethnobotanical information comprised the local name, source, part(s) used, method of traditional preparation, and demographic information of the informants such as age, gender, experience, and educational background.

The ethical approval was obtained from the Ethical Review Committee, Faculty of Medicine, University of Ruhuna, Sri Lanka (permit issued on 15/09/2014). Data were analyzed using SPSS Statistics package version 20.

### 2.3. Plant Specimen Collection and Preservation

Plant species used for the treatment of inflammatory conditions were collected, dried, preserved, and mounted on herbarium sheets. Botanical names and families were verified using the book series titled “Flora of Sri Lanka” by one of the authors (MTN), who is a botanist. The botanical names have also been checked with the data available at http://www.theplantlist.org. The specimens were deposited at the Herbarium in the Department of Biochemistry, Faculty of Medicine, University of Ruhuna, Sri Lanka.

### 2.4. Quantitative Analysis of the Ethnobotanical Information

The knowledge of medicinal plant usage was quantitatively assessed using the relative frequency of citation (RFC), family importance value (FIV), and use value (UV). The RFC and FIV were calculated to quantitatively determine the consensus between informants on the use of medicinal plants in the region as it gives the local importance of a species or a family. The RFC was calculated using the standard method of Vitalini et al. [11] and Savikin et al. [12]:

(1) $$\begin{array}{c} \text{RFC}=\frac{\text{FC}}{N}\left(0<\text{RFC}<1\right). \end{array}$$

The value of RFC for species and families of medicinal plants is based on the citing percentage of informants for that particular species and plant family. FC is the number of informants who mentioned the species, while *N* is the total number of informants participating in the study.

The family importance value (FIV) was calculated by taking the percentage of informants mentioning the family:

(2) $$\begin{array}{c} \text{FIV}=\frac{\text{FC}\left(\text{family}\right)}{N}\times 100\text{,} \end{array}$$

where FC is the number of informants mentioning the family, while *N* is the total number of informants participating in the study.

The use value demonstrates the relative importance of plant species known locally and was determined by the following formula [13, 14]:

(3) $$\begin{array}{c} UV_{i}=\sum \frac{U_{i}}{N_{i}}, \end{array}$$

where *U*_*i*_ is the number of use reports described by each informer for species *i*, while *N*_*i*_ is the total number of informers describing the specific species *i*.

## 3. Results

Out of the total participants, 232 (50.7%) claimed the use of medicinal plants for the treatment of inflammatory conditions such as fever, cough, asthma, joint pain, and swellings. 46.16% have mentioned the reason for their choice as their belief in the safety and low adverse effects associated with the herbal formulations. The majority of the users (65.91%) claimed that they use these herbal preparations at the initial stage of a disease before using any other medications, while 18.94% have mentioned the simultaneous usage of other medications. A considerable proportion (12.12%) claimed that the herbal therapeutics will be used as a last resort, when other treatment methods have failed. These people diagnose the inflammatory conditions by their signs and symptoms rather than specific laboratory tests. The knowledge of the herbal remedies had transferred through generations, while the media have also contributed in promoting the usage of herbal therapeutics (Table 1).

**Figure 2:**
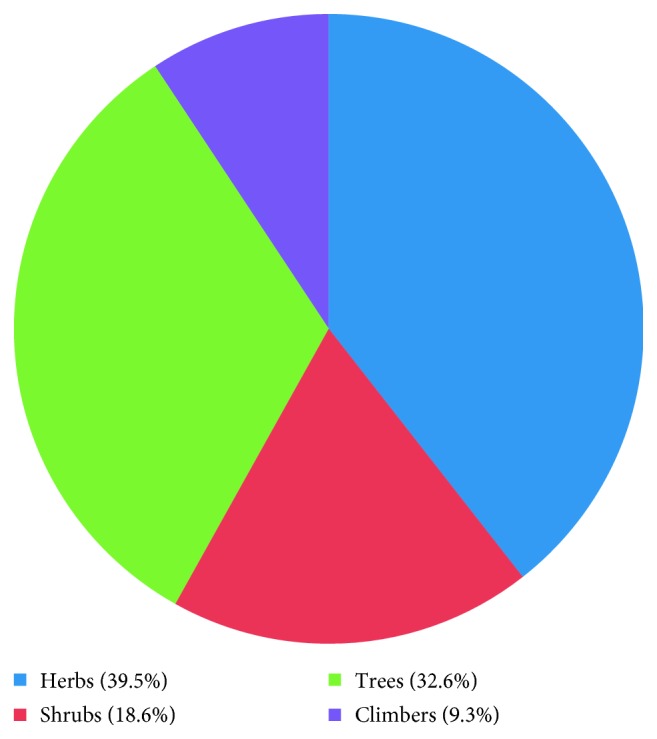
Life form of the plants used as anti-inflammatory remedies.

**Figure 3:**
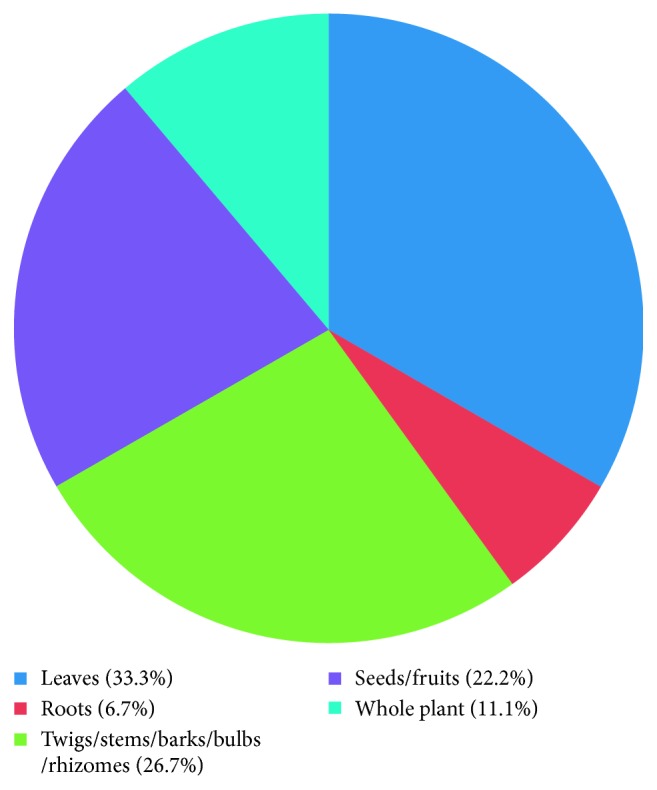
Plant parts used in herbal preparations.

**Figure 4:**
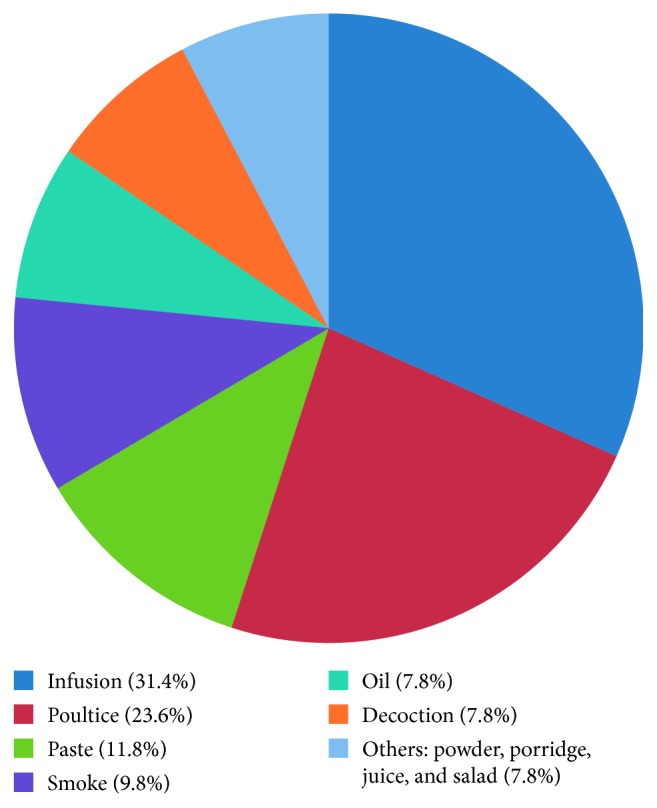
Mode of utilization reported to treat inflammatory conditions.

A total of 43 medicinal plants belonging to 28 plant families were mentioned, out of which *Coriandrum sativum* (RFC = 0.23) was the most cited species, followed by *Coscinium fenestratum* (RFC = 0.13) and *Adhatoda vasica* (RFC = 0.12). The most cited plant family was Fabaceae, and the family importance value was highest in Apiaceae (23.58%) (Table 2). The most dominant life form of the species reported includes herbs (39.5%) (Figure 2). The most frequently used plant part was leaves (33.3%) (Figure 3), followed by twigs/stems/barks/bulbs/rhizomes (26.7%). Medicinal plants used in folk herbal remedies were prepared and administered in various forms, and the most common preparation method was infusion (31.4%) (Figure 4). The percentage of oral administration (47%) of herbal preparation was almost higher than that of external or topical application (43.2%) and inhalation (9.8%). Most of the crude drugs were prepared from single plant species; however, combinations of multiple species were also reported, while some preparations were administered along with honey, sugar, sugar candy, salt, coconut oil, and so on. Although various commercial preparations of herbal origin were mentioned by some of the participants, these were not considered during the data analysis. The summary of the medicinal plant species used in Gampaha District to treat inflammatory conditions is given in Table 3.

Majority of the people belonging to the nonuser category (70.9%) had used some kind of herbal therapeutics at some stage of their lives and mentioned that the usage was discontinued due to the difficulty in preparation (24.69%) and collection of plant materials (22.22%). Other reasons that hindered the usage of herbal preparations have been identified as a relatively long period of time taken for healing and the unpleasant smell and the taste. Some of these people have mentioned that they do not have any faith as the effectiveness of the herbal formulations is not scientifically proven. Interestingly, 71.43% of the nonusers mentioned that they would shift to herbal products if the efficacy of these products could be scientifically validated.

## 4. Discussion

Sri Lanka has a rich source of medicinal plants distributed in different geographical regions, and a large section of the Sri Lankan population still rely on traditional plant medicines that are abundantly available, economical, and believed to be of little or no side effects. Indigenous knowledge of the remedies has been transferred from one generation to the next through traditional healers, knowledgeable elders, or ordinary people, mostly without any written documents. However, factors such as cultural change, particularly the influence of modernization, lack of written documents, deforestation, environmental degradation, and lack of interests shown by the younger generations impose a serious threat to the enhancement of existing knowledge and practices of medicinal plants. Thus, ethnobotanical studies and subsequent conservation measures are urgently required to prevent the loss of valuable indigenous knowledge of medicinal plants. Furthermore, the importance of ethnobotanical research has been increasing, since potential sources of drugs could disappear in the future as a result of the rapid loss of biodiversity. This is the first report of an in-depth ethnobotanical study in Sri Lanka, and it enabled us to make some contribution in the preservation of the traditional systems of medicine by proper documentation and identification of specimens.

Owing to the high global prevalence of pathophysiological conditions linked with inflammation, a substantial number of ethnobotanical studies have specifically been focused on assessing the indigenous knowledge of traditional anti-inflammatory remedies. The study conducted in five local government areas in Ogun State of Nigeria has led to the identification of 83 different plant species that have been used in the management of inflammatory diseases [15]. A total of 34 species in 32 genera and 22 families were encountered during the field study conducted to gather ethnobotanical information on traditional medicinal plants exclusively used for the management of inflammation-related ailments by the Khampti community of Arunachal Pradesh, India [16]. Despite the wide utility of medicinal plants for the treatment of inflammatory conditions, surprisingly, no such studies have been conducted in Sri Lanka yet. Furthermore, there is a rich reserve of indigenous knowledge of medicinal plants due to a large number of practitioners of traditional medicine; however, the scientific validation of theses traditional claims is still at its infancy.

The study revealed that the medicinal uses of some of the plant species mentioned by the participants have not been documented in the literature, for example, the usage of *Calotropis gigantea* for sprains/swellings and *Strychnos potatorum* for swellings in the joints. The medicinal plants with a higher frequency of citation as determined from the current study would signpost the probable existence of valuable phytochemical compounds, and it requires a search for prospective new drugs to cure many inflammatory disorders. Therefore, the effectiveness and the safety of the identified plants will be assessed by phytochemical and pharmacological investigations in the follow-up studies. Therefore, the present study based on indigenous knowledge of medicinal plants would contribute towards the national development agenda of the country, a subarea of the National Research and Development Framework (NRDF) in Sri Lanka [17].

However, there are certain limitations of the current study, which need to be improved to conduct a more comprehensive island-wide study in the future. For example, when the households were selected randomly from a list, households with strong beliefs on herbal medicine could have been overlooked as well as households who profusely refuse such remedies. Furthermore, only the people who can speak English or Sinhala have been recruited to this study; therefore, it may not represent the whole population of the country. If the above limitations could be overcome, then it would enable the documentation and preservation of indigenous knowledge of herbal medicine restricted among different segments of the Sri Lankan population.

## 5. Conclusion

This study reports the first ethnobotanical survey in Sri Lanka. Among 43 medicinal plants belonging to 28 reported plant families, Fabaceae was the most cited plant family. The most popular medicinal plants among the inhabitants in Gampaha District include *Coriandrum sativum*, *Coscinium fenestratum*, and *Adhatoda vasica*. The investigations revealed that the indigenous herbal medicines are still common among the local communities, and even the nonusers are ready to shift to herbal products if systematic scientific information is available. Therefore, the present study signifies the necessity of the scientific validation of herbal remedies.

## Figures and Tables

**Table 1:** Statistics on the usage of herbal therapeutics as anti-inflammatory remedies.

Parameter	Percentage (%)
1. Demographic data of regular users	
1.1. Gender	
Male	39.28
Female	60.72
1.2. Age group (years)	
30–45	39.17
46–60	44.17
61–75	8.33
>75	8.33
1.3. Educational background	
University degree/diploma and above	12.18
Up to university entrance exam^*∗*^	37.39
Ordinary-level education and below^*∗∗*^	46.95
No schooling	3.48

2. Source of information/knowledge	
From ancestors/family members	60.42
Neighbours/friends	13.89
Doctors/traditional physicians	7.64
Media	13.19
Own experience	4.86

3. Reason for usage	
Safe/less side effects	46.16
Previous success	35.67
Easy access to the plant materials	6.29
High cost of other treatment methods	3.49
Nonavailability of modern health care facilities	0
Failure of other treatment methods	8.39

^*∗*^The highest educational qualification which can be obtained at school (13 years of school education); ^*∗∗*^1–11 years of school education.

**Table 2:** Family importance value (FIV).

Family	FIV
Acanthaceae	11.35
Amaryllidaceae	0.22
Apiaceae	23.58
Asclepiadaceae	0.22
Asphodelaceae	0.44
Bignoniaceae	0.87
Clusiaceae	0.44
Combretaceae	0.44
Crassulaceae	0.22
Cyperaceae	0.44
Euphorbiaceae	1.31
Fabaceae	3.49
Lamiaceae	0.44
Lauraceae	0.65
Loganiaceae	0.44
Malvaceae	0.87
Meliaceae	0.44
Menispermaceae	13.53
Molluginaceae	0.22
Moringaceae	0.87
Piperaceae	1.31
Poaceae	0.44
Rutaceae	13.32
Sapindaceae	0.22
Sapotaceae	0.87
Solanaceae	10.92
Verbenaceae	3.49
Zingiberaceae	12.23

**Table 3:** Medicinal plant species used in Gampaha District to treat inflammatory conditions.

Family	Scientific name	Vernacular name	Life form	Parts used	Preparation	Inflammatory conditions treated	RFC	UV	Reported usage in the literature [6]	Voucher specimen number
Acanthaceae	*Adhatoda vasica* Nees	Adhathoda	Shrub	Leaves, twigs, roots	Infusion, poultice	Swellings in joints, cough, asthma, catarrh	0.12	2.56	Diarrhea, fever, asthma	MNWP-01
Amaryllidaceae	*Allium sativum* L.	Sudulunu	Herb	Bulbs	Infusion	Asthma	0.002	1.0	Asthma, gout	MNWP-02
Apiaceae	*Coriandrum sativum* L.	Koththamalli	Herb	Seeds	Infusion	Cold, fever, asthma	0.23	2.59	Inflammation	MNWP-03
Asclepiadaceae	*Calotropis gigantea* (L.) Dryand.	Wara	Shrub	Whole plant	Poultice	Sprains, swellings	0.002	1.0	Skin diseases, leprosy, jaundice, sinus troubles	MNWP-04
Asphodelaceae	*Aloe vera* (L.) Burm.f.	Komarika	Herb	Leaves	Paste	Cough, asthma	0.004	1.0	Cough	MNWP-05
Bignoniaceae	*Oroxylum indicum* (L.) Kurz	Thotila	Tree	Barks	Poultice	Swellings in joints	0.009	1.0	Rheumatic swellings, fractures	MNWP-06
Clusiaceae	*Calophyllum inophyllum* L.	Domba	Tree	Seeds	Oil, poultice	Swellings in joints	0.004	1.0	Chronic rheumatism, swellings in joints	MNWP-07
Combretaceae	*Terminalia chebula* Retz.	Aralu	Tree	Fruits	Powder	Fever	0.004	1.0	Fever, eye diseases, piles, chronic dysentery	MNWP-08
Crassulaceae	*Kalanchoe laciniata* (L.) DC.	Akkapana	Herb	Leaves	Infusion	Cough, asthma, cold	0.002	1.5	Urinary diseases, diarrhea, dysentery, cough, cold	MNWP-09
Cyperaceae	*Cyperus rotundus* L.	Kaladuru	Herb	Whole plant	Infusion	Fever	0.004	1.0	Fever, bronchitis	MNWP-10
Euphorbiaceae	*Phyllanthus emblica* L.	Nelli	Tree	Fruits	Poultice	Redness and swellings in the eye	0.004	1.0	Inflammation in the eye, gonorrhea, diarrhea, urinary diseases	MNWP-11
	*Ricinus communis* L.	Enderu	Shrub	Leaves	Poultice	Headache, joint pains, swellings	0.009	1.5	Headache, boils, rheumatism	MNWP-12
Fabaceae	*Glycyrrhiza glabra* L.	Welmi	Herb	Twigs	Infusion	Catarrh, asthma	0.022	1.4	Laryngitis, bronchitis	MNWP-13
	*Pterocarpus santalinus* L.f.	Rath-handun	Tree	Stems	Paste	Headache, pain, swellings in joints	0.004	1.5	Rheumatism, insect bites, headache	MNWP-14
	*Tamarindus indica* L.	Siyabala	Tree	Leaves	Paste	Swellings in joints	0.006	1.0	Boils, rheumatism	MNWP-15
	*Desmodium triflorum* (L.) DC.	Undupiyali	Herb	Leaves	Paste	Swellings in joints	0.004	1.0	Ulcers, dysentery, inflammation	MNWP-16
	*Trigonella foenum-graecum* L.	Uluhal	Herb	Seeds	Poultice	Pain in joints	0.002	1.0	Dyspepsia, diarrhea, rheumatism	MNWP-17
Lamiaceae	*Plectranthus zeylanicus* Benth.	Iriweriya	Herb	Roots	Infusion, decoction	Fever	0.004	1.0	Fever, cough, asthma	MNWP-18
Lauraceae	*Cinnamomum camphora* (L.) J.Presl	Kapuru	Tree	Fruits	Oil	Fever, swellings in joints, asthma	0.002	1.5	Inflammation, bruises, sprains, whooping cough, asthma	MNWP-19
	*Litsea glutinosa* (Lour.) C.B.Rob.	Bomi	Tree	Barks	Paste	Swellings in joints	0.004	1.0	Diarrhea, dysentery, sprains, bruises, rheumatism	MNWP-20
Loganiaceae	*Strychnos potatorum* L.f.	Ingini	Tree	Seeds	Paste	Swellings in joints	0.004	1.0	Eye diseases, diarrhea	MNWP-21
Malvaceae	*Sida acuta* Burm.f.	Babila	Herb	Roots	Infusion, decoction	Fever	0.009	1.0	Fever, impotency, rheumatism	MNWP-22
Meliaceae	*Azadirachta indica* A.Juss.	Kohomba	Tree	Leaves	Poultice	Pain in joints	0.004	1.0	Catarrh, leprosy and skin diseases, rheumatism, wounds, ulcers	MNWP-23
Menispermaceae	*Coscinium fenestratum* (Goetgh.) Colebr.	Veniwelgata	Woody climber	Stems	Infusion	Fever, cough, asthma	0.13	2.43	Fever, tetanus, dressing wounds, ulcers	MNWP-24
	*Tinospora cordifolia* (Willd.) Miers	Rasakida	Climber	Stems	Infusion	Fever	0.009	1.0	Fever, skin diseases, diabetes, dysentery, rheumatism	MNWP-25
Molluginaceae	*Mollugo cerviana* (L.) Ser.	Pathpadagum	Herb	Whole plant	Infusion	Fever, asthma	0.065	1.63	Fever, skin diseases, gonorrhea	MNWP-26
Moringaceae	*Moringa oleifera* Lam.	Murunga	Shrub	Barks	Infusion, poultice	Asthma, swellings in pain	0.009	1.5	Asthma, gout, rheumatism, remedy for snakebite poisoning	MNWP-27
Piperaceae	*Piper longum* L.	Thippili	Herb	Fruit	Infusion, decoction	Fever, asthma, cough	0.013	1.3	Fever, cough, bronchitis	MNWP-28
Poaceae	*Eleusine indica* (L.) Gaertn.	Bela-tana	Herb	Whole plant	Poultice	Swellings, sprains	0.004	1.5	Sprains and dislocations	MNWP-29
Rutaceae	*Aegle marmelos* (L.) Corrêa	Beli	Tree	Leaves, roots	Decoction	Asthma, fever	0.009	1.5	Fever, asthma, dysentery, piles, dyspepsia	MNWP-30
	*Atalantia ceylanica* (Arn.) Oliv.	Yakinarang	Shrub	Leaves	Infusion, smoke	Cough, cold, breathing difficulties, asthma	0.01	2.36	Catarrh, bronchitis and other chest complaints, fever	MNWP-31
	*Citrus aurantium* L.	Embul-Dodam	Tree	Fruits	Juice	Cough, to draw out phlegm	0.026	1.25	Chronic cough	MNWP-32
	*Citrus aurantifolia* (Christm.) Swingle	Dehi	Tree	Leaves	Smoke	Cough, headache	0.08	1.11	Cough, stomachache, cleaning wounds, dysentery	MNWP-33
Sapindaceae	*Cardiospermum halicacabum* L.	Wel-penela	Climber	Whole plant	Infusion	Swellings in joints	0.002	1.0	Rheumatism, dropsy, earache, bronchitis	MNWP-34
Sapotaceae	*Madhuca longifolia* (J.Koenig ex L.) J.F.Macbr.	Mee	Tree	Seeds	Oil, poultice	Swellings and pain in joints	0.009	1.5	Fractures, rheumatism, snakebites	MNWP-35
Solanaceae	*Solanum xanthocarpum* Schrad. & H. Wendl.	Katuwelbatu	Herb	Leaves	Infusion	Fever, cough, asthma	0.087	2.22	Cough, asthma, colic fever, toothache	MNWP-36
	*Solanum surattense* Burm. f.	Ela-batu	Herb	Leaves	Porridge, smoke	Cough, asthma	0.013	1.67	Rheumatism, cough, diarrhea	MNWP-37
	*Solanum trilobatum* L.	Wel-Thithbatu	Shrub	Leaves	Porridge, salad	Prolonged cough	0.009	1.0	Cough	MNWP-38
Verbenaceae	*Lantana camara* L.	Gandapana	Shrub	Leaves	Smoke	Fever, cough, asthma	0.009	2.25	Asthma, fever, cough	MNWP-39
	*Vitex negundo* L.	Nika	Shrub	Leaves	Smoke, paste	Swellings in joints, cough, asthma, fever	0.026	1.92	Rheumatic swellings, headache, catarrh, asthma	MNWP-40
Zingiberaceae	*Alpinia galanga* (L.) Willd.	Araththa	Herb	Rhizome	Infusion	Fever	0.026	1.0	Rheumatism, bronchitis	MNWP-41
	*Zingiber officinale* Roscoe	Inguru	Herb	Rhizomes	Infusion	Fever, asthma, cough	0.092	1.69	Cold, cough, fever, asthma	MNWP-42
	*Curcuma zedoaria* (Christm.) Roscoe	Haran-kaha	Herb	Rhizomes	Poultice	Swellings in joints	0.004	1.0	Sprains, dermatitis, wound healing	MNWP-43

## Abbreviations

RFC: Relative frequency of citation
FIV: Family importance value
UV: Use value.
